# Plant Glycosides and Glycosidases: A Treasure-Trove for Therapeutics

**DOI:** 10.3389/fpls.2020.00357

**Published:** 2020-04-07

**Authors:** Kassiani Kytidou, Marta Artola, Herman S. Overkleeft, Johannes M. F. G. Aerts

**Affiliations:** ^1^Department of Medical Biochemistry, Leiden Institute of Chemistry, Leiden University, Leiden, Netherlands; ^2^Department of Bio-organic Synthesis, Leiden Institute of Chemistry, Leiden University, Leiden, Netherlands

**Keywords:** plant glycosides, carbohydrate processing enzymes, glycosidases, glycosylation, enzyme replacement therapy (ERT), plant production platforms, glycosphingolipids, glycosidase activity-based probes

## Abstract

Plants contain numerous glycoconjugates that are metabolized by specific glucosyltransferases and hydrolyzed by specific glycosidases, some also catalyzing synthetic transglycosylation reactions. The documented value of plant-derived glycoconjugates to beneficially modulate metabolism is first addressed. Next, focus is given to glycosidases, the central theme of the review. The therapeutic value of plant glycosidases is discussed as well as the present production in plant platforms of therapeutic human glycosidases used in enzyme replacement therapies. The increasing knowledge on glycosidases, including structure and catalytic mechanism, is described. The novel insights have allowed the design of functionalized highly specific suicide inhibitors of glycosidases. These so-called activity-based probes allow unprecedented visualization of glycosidases cross-species. Here, special attention is paid on the use of such probes in plant science that promote the discovery of novel enzymes and the identification of potential therapeutic inhibitors and chaperones.

## Introduction

### Plant Metabolites and Their Glycosylation

Plants provide nutrition and the human body has evolved to thrive optimally on this nourishment. Besides the nutritional value, plant-derived food influences the microbiome in the gastrointestinal tract with physiological effects (Theilmann et al., 2017). Plants produce a huge variety of secondary metabolites that can be decorated with sugars, i.e., glycosylated (Jones and Vogt, 2001; Gachon et al., 2005; Wink, 2015). Specific plant glycosyltransferases (GTs) using nucleotide-sugars as donors can attach specific sugar moieties to an acceptor molecule (Henrissat and Davies, 2000; Jones and Vogt, 2001). Glycosyl hydrolases, so-called glycosidases, remove specific sugar moieties. Most of these enzymes are retaining exo-glycosidases (Coutinho et al., 2003). Some of these glycosidases are also able to synthetically transglycosylate in the presence of high concentrations of an acceptor molecule, a reaction implying the transfer of a sugar moiety from a substrate to an acceptor molecule (Morant et al., 2008).

Glycosylation of metabolites in plants serves multiple purposes. Upon glycosylation, hydrophobic metabolites become more water-soluble which improves their bio-distribution and metabolism (Kren and Martinkova, 2001; Pandey et al., 2014). Increased solubility and amphiphilicity of glycosylated metabolites may assist their transport across cell membranes (Vetter, 2000). The attachment of sugars to small metabolites raises their molecular weight and melting point. This allows synthesis and storage of precursors of volatile compounds that can be released on demand after hydrolysis (Ohgami et al., 2015). The stability of glycosylated metabolites may depend on the position where the sugar moiety is attached, for example the 6-*O*-glucosides of ascorbic acid are chemically less stable than the 2-*O*-glucoside form or non-glycosylated ascorbic acid (Jones and Vogt, 2001). Furthermore, detoxification of harmful molecules can take place through glycosylation. Glycosylation may generate a non-toxic agent that later can be re-activated and used as aglycone in defense against parasites and plant-eating organisms such as herbivores. Examples are cyanogenic glycosides produced by plants. These consist of an α-hydroxynitrile group attached to a sugar moiety, often a D-glucose (Vetter, 2000). Release of cyanohydrin aglycone leads to spontaneous transformation to the corresponding ketone or aldehyde and release of hydrogen cyanide (prussic acid) (Cressey and Reeve, 2019). Hydrolysis of cyanogenic glycosides can be mediated by endogenous β-glucosidases when brought into contact with the substrate upon damaging of plant cells (Cressey and Reeve, 2019). Additionally, hydrolysis may take place by the gut microbiome during digestion of plant material. The first identified cyanogenic glycoside was amygdalin isolated from almonds in 1830 (Robiquet and Boutron-Charlard, 1830). Cyanogenic glycosides are ubiquitous in plants, being identified in more than 2,500 species (Vetter, 2000). The sugars attached to the aglycone may vary from a disaccharide to monosaccharide, usually glucose (Vetter, 2000; Haque and Bradbury, 2002; Cressey and Reeve, 2019). Cassava, *M. esculenta*, produces the cyanogenic glycosides linamarin and lotaustralin and consumption may cause severe pathology (Kamalu, 1991; Kamalu, 1993). Finally, another example of regulating biological activity by glycosylation is provided by glycosylated phytohormones such as abscisic acid (ABA), auxin (IAA), cytokinins (CKs), brassinosteroids (BRs), salicylic acid, and gibberellin that regulate growth, development, and responses to environmental stresses (Gachon et al., 2005). Glycosylation of phytohormones usually leads to inactive storage forms of plant hormones that can be hydrolyzed for activation, allowing rapid responses and maintaining the hormonal homeostasis (Kren and Martinkova, 2001; Stupp et al., 2013; Pandey et al., 2014).

In this review, we pay attention to the natural occurrence of glycosides in plants with emphasis to glycolipids and touch upon their metabolizing enzymes. We address the use of plant lipids for therapeutic purposes as well as their potential harmful effects. Described is the increasing use of plants as production platforms for therapeutic enzymes, in particular glycosidases for the treatment of lysosomal storage disorders. Finally, we discuss the recent design of unprecedented tools to study glycosidases, cross-species. These so-called activity-based probes (ABPs) are modified cyclophellitols that allow *in situ* visualization of their target glycosidases. ABPs label glycosidases cross-species due to the highly conserved catalytic pockets and find many applications like discovery of glycosidases in several organisms, diagnosis of inherited lysosomal glycosidase deficiencies, visualization of tissue distribution and subcellular localization of endogenous and exogenous (therapeutic) glycosidases and the identification of therapeutic inhibitors and chaperones.

## Beneficial Glycosylated Plant Metabolites

### Plant-Derived Agents and Human Health

Balanced consumption of vegetables is nowadays in the center of attention, particularly prompted by the worldwide epidemic of obesity and associated health problems. There is considerable interest in plant products from practitioners of regular medicine and pharmaceutical industry. Of note, the first generation of pharmaceuticals largely consisted of plant-derived products or minor chemical modifications thereof (Friend, 1974). The longstanding popularity of natural plant products with alternative medicine advocates stems in many cases from ancient use of such materials in traditional medicine.

The chemical structure of plant glycosides determines their biological action(s) and bioavailability (uptake). In this respect, attention is first paid to glycosylated flavonoids.

#### Glycosylated Flavonoids

The predominant polyphenols in food (i.e., fruits, vegetables, nuts) and beverages (i.e., tea, wine) are flavonoids (Pandey and Rizvi, 2009; Pan et al., 2010). Plant flavonoids can be categorized into subclasses: flavonols, isoflavonols, flavones, flavanones, flavanols (catechins), and anthocyanidins (Ross and Kasum, 2002; Xiao et al., 2014). Daily consumption of several milligrams of flavonoids (25 mg to 1 g/day) is common (Hertog et al., 1993; Tsuda et al., 1999; Ross and Kasum, 2002).

**FIGURE 1:**
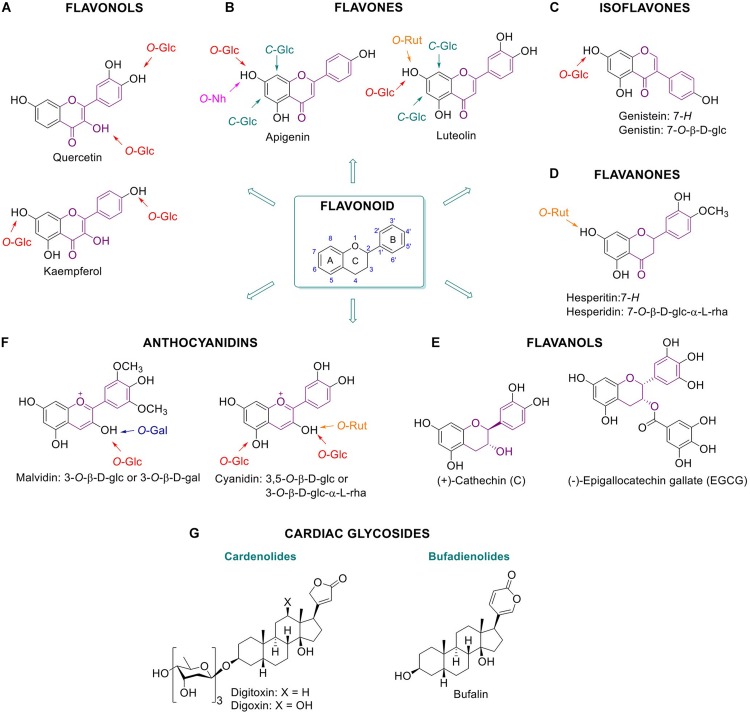
Glycosylated plant metabolites beneficial for humans. Flavonoids and some of their glycoside metabolites: flavonols **(A)**, flavones **(B)**, isoflavones **(C)**, flavanones **(D)**, flavanols **(E)**, and anthocyanidins **(F)**. Chemical structures of cardiac glycosides **(G)**. Bufalin is an animal-derived cardiac glycoside.

Many plant **flavonoids** (see Figure 1 for general structures) are glycosylated (Day et al., 1998; Tohge et al., 2017). Glycosides are linked to the phenolic hydroxyls, via α- or β-D-glycosidic linkages (Murota and Terao, 2003). This type of modification may involve a single oligosaccharide or in some cases a polysaccharide moiety (Xiao et al., 2014). Commonly reported benefits of flavonoid glycosides are anti-oxidants and anti-inflammatory activities which find application in prevention and disease management (Lin and Harnly, 2007; Xiao et al., 2014). To illustrate this, some examples of each subclass are here discussed.

**Flavonols** are characterized by a phenolic substitution at position 2 of its 3-hydroxyflavone backbone. Quercetin is a flavonol present in plants, fruits and vegetables. It can occur as diverse glucosylated forms: for example quercetin-4′-*O*-β-D-glucoside or quercetin-3,4′-*O*-β-D-glucoside are predominant in onion with the glucose attached at the 3 or/and 4′ position of the phenol respectively (Murota and Terao, 2003) (Figure 1A). It has been suggested that phenolic hydroxyl groups contribute to the free radical scavenging activity of the molecules (Bors et al., 1990). The health benefits of quercetin are generally attributable to its anti-oxidant action positively impacting on glutathione and reactive oxygen species (ROS) (Xu et al., 2019). The anti-oxidant action is in part mediated by the modulation of enzymes associated with oxidative stress like acetylcholinesterase and butyrylcholinesterase (Xu et al., 2019). Kaempferol, present in broccoli, apples, tea, strawberries, and beans, is another flavonol with reported antioxidant and anti-inflammatory properties (Chen and Chen, 2013).

Whereas flavonols have an OH group at C-3, the **flavones** bear a hydrogen in that position (Figure 1B). Apigenin and luteolin are *flavones* found in plant food. Apigenin occurs in a wide variety of vegetables and fruits such as parsley, celery, chamomile, oranges, thyme, onions, honey, and spices, as well as beverages derived from plants such as tea, beer, and wine. It exists largely as *C*-glycosylated form, being more stable and reactive than the *O*-glycosylated counterparts. Apigenin is found to be absorbed as glycosylated structure and to exert antioxidant, anti-inflammatory and anti-cancer effects. For instance, glycosylated forms of apigenin with various pharmacological activities are apigenin 6-*C*-glucoside (isovitexin), apigenin 8-*C*-glucoside (vitexin), apigenin 7-*O*-glucoside, and apigenin 7-*O*-neohesperidoside (rhoifolin) (He et al., 2016). Luteolin, present in carrots, peppers, celery, olive oil, peppermint, thyme, rosemary and oregano, is reported to have antioxidant effects and it is assumed to inhibit angiogenesis, induce apoptosis and thereby prevent carcinogenesis *in vivo* (Lin et al., 2008). Well known glycosylated forms of luteolin in citrus fruits are luteolin 7-*O*-rutinoside and lucenin-2 (luteolin 6,8-di-*C*-glucoside). Furthermore, cynaroside, the 7-*O*-glucoside derivative of luteolin, is found in *Lonicera japonica Thunb.* and *Angelica keiskei*. and also shows anti-oxidant and anti-inflammatory activity (Lin et al., 2008; Lopez-Lazaro, 2009; Chen et al., 2012; Nho et al., 2018).

**Isoflavones** bear a phenolic moiety at position 3 instead of 2 (Figure 1C). Genistein, an isoflavone found predominantly in soy, and together with its glycosylated form genistein, is reported to provide multiple health benefits. Several studies demonstrated that genistein has anti-diabetic effects, in particular through direct positive effects on β-cells and glucose-stimulated insulin secretion. In addition, protection against apoptosis is reported, independent of its function as an estrogen receptor agonist, antioxidant action, and inhibition of tyrosine kinase activity (Fotsis et al., 1993; Record et al., 1995; Allred et al., 2001; Pandey et al., 2014).

**Flavanones** are characterized by a saturated C2–C3 bond in the C ring and normally occur as a racemic mixture (Figure 1D). Hesperidin, a 7-*O*-rutinoside flavone, is a natural product with a wide range of biological effects, in particular it presents inhibitory effect against the development of neurodegenerative diseases (Hajialyani et al., 2019). Hesperidin, a dietary **flavanone**, and its aglycone hesperetin, are found predominantly in citrus fruits such as oranges and lemons. These compounds are considered to exert beneficial anti-inflammatory and anti-oxidative action (De Souza et al., 2016).

**Flavanols** (a.k.a. catechins) have a 2-phenyl-3,4-dihydro-2*H*-chromen-3-ol skeleton and are mainly found in tea. Contrary to other flavonoids, flavanols are often not glycosylated and their glycosylation normally decreases anti-oxidant activity (Plumb et al., 1998). The beneficial effects of green tea have been attributed to its high content of polyphenolic catechins, including catechin (C), (−)-epicatechin (EC), (−)-epigallocatechin (EGC), (−)-epicatechin-3-gallate (ECG), and (−)-epigallocatechin-3-gallate (EGCG) (Figure 1E). Among them, EGCG is the polyphenolic catechin with the highest antioxidant effect as ROS scavenger and metal ion chelator, and it finds application in prevention of disease caused by oxidative stress, such as cancer, cardiovascular diseases, neurodegenerative disease, neuropathic pain, and diabetes (Roghani and Baluchnejadmojarad, 2009; Xifró et al., 2015).

**Anthocyanidins** possess a 2-phenylchromenylium ion backbone and are the deglycosylated version of anthocyanins (Figure 1F). Anthocyanins are abundant pigments in many red berries with documented antioxidant action. Examples are cyanidin-3-*O*-rutinoside and cyanidin-3,5-*O*-diglucoside (Feng et al., 2016). Likewise, anti-inflammatory properties are reported for the anthocyanidin malvidin-3-*O*-β-D-glucoside and malvidin-3-*O*-β-D-galactoside in blueberries by blocking the NF-κB pathway mechanism (Huang et al., 2014).

To which extent glycosylation of flavonoids contributes to their beneficial action is not always well understood. Glycosylation of flavonoids might favor bioavailability and uptake into the body. One advantage of glycosylation is that it can stabilize the molecules, preserving their structural integrity and therefore enabling their accumulation. In addition, glycosylation serves as a transport signal among the different compartments of the plant cell. For example, cyanogenic glucosides are transported only in their glycosylated form (Jones and Vogt, 2001). Flavonoid glycosides may be converted to their aglycones prior to absorption by intestinal epithelial cells. However, some glycosylated flavonoids are apparently also absorbed as such (for example, cyanidin-3-*O*-β-D-glucoside and glycosylated apigenin) (Murota and Terao, 2003; Xiao et al., 2014, 2016). The linked sugar moiety, the type of linkage (*O*- versus *C*-) and the position of the glycoside attachment may influence the bioactivity of a flavonoid. An example of the latter forms the inferior free radical scavenging of quercetin-4′-*O*-β-D-glucoside compared to quercetin-3-*O*-β-D-glucoside (Yamamoto et al., 1999). This difference is due to the fact that the flavonoids’ free radical scavenging activity depends on phenolic hydroxyl groups which act as electron donors. In particular, a catechol moiety with two neighboring hydroxyls has high electron donation ability (Murota and Terao, 2003). The antioxidant activities of glycosylated flavonoids can partly be also attributed to their chelation action, with the catechol group also playing an key role in the process (Murota and Terao, 2003). Interestingly, *C*-glycosylation enhances some of the beneficial traits of flavonoids such as their antioxidant and anti-diabetic activities. *O*-Glycosylation is reported to reduce flavonoid bioactivity and absorption (Hostetler et al., 2012; Xiao et al., 2014).

The anti-inflammatory effects of flavonoids can be attributed to reduction of cytokine-induced inflammation by the inhibition of tumor necrosis factor-α (TNF-α) signaling and reduced expression of pro-inflammatory genes by down-regulation of NF-κB (Ramos, 2007; Pan et al., 2010; Huang et al., 2014). An example is provided by the anti-inflammatory flavonol kaempferol which is present in broccoli, tea and vegetables. During osteoporosis, pro-inflammatory cytokines, e.g., TNF-α, are expressed and cause bone disruption and further cytokine production. Kaempferol antagonizes the TNF-α induced production of interleukin-6 (IL-6) and monocyte chemotactic protein-1 (MCP1a), as well as the RANKL triggered osteoclast precursor cell differentiation (Pang et al., 2006; Pan et al., 2010). Another example is the anti-inflammatory effect of glycosylated anthocyanins present in blueberries, malvidin-3-*O*-glucoside and malvidin-3-*O*-β-D-galactoside. These molecules reduce the levels of MCP1, intercellular adhesion and vascular cell adhesion molecule-1 at protein and mRNA level in endothelial cells through the inhibition of TNF-α. In addition, they block the NF-κB pathway by affecting IκBα degradation and the nuclear translocation of p65 (Huang et al., 2014).

For many flavonoids miscellaneous anti-cancer effects have been reported. The presumed modes of action of flavonoids as anti-cancer agents are diverse and the role of glycosylation in such anti-tumor effect is often not well understood. Examples of flavonoids with reported anti-tumor action are kaempferol (Ramos, 2007; Chen and Chen, 2013), peonidin 3-*O*-β-D-glucoside, genistein, genistin, and EGCG (Fotsis et al., 1993; Record et al., 1995; Allred et al., 2001; Pandey et al., 2014; Xifró et al., 2015). Genistein and genistin are, however, also reported to stimulate breast cancer cells *in vivo* at very low concentrations (nM range), acting as estrogen agonists in mice mammary glands (Allred et al., 2001). Daidzin of soybeans is another well-studied isoflavone 7-*O*-β-D-glucoside with similar anti-cancer properties as genistein. Anti-cancer action has also been documented during the last decades for apigenin, hesperidin and its aglycone hesperetin (Madunić et al., 2018).

**Plant-derived cardiac glucosides** are secondary metabolites consisting of a steroid backbone functionalized with a lactone ring at the 17-β position and a sugar moiety at the 3-β position (Figure 1G). Cardiac glycosides can be classified as cardenolides or bufadienolides depending on the 5- or 6-membered lactone ring, respectively. Cardenolides are known since ancient times for their positive effects on cardiac arrhythmia, congestive heart failure and atrial fibrillation (Nesher et al., 2007). Their main role as antiarrhythmic agents is based on their ability to inhibit the Na+/K+ ATPase ion pump, thus increasing intracellular potassium concentrations (Kelly, 1990; Kepp et al., 2012; Patel, 2016). In response to this, intracellular calcium increases which promotes more efficient myocardial contraction and improves cardiac pump activity (Newman et al., 2008). Well known examples of therapeutic cardiac glycosides are digoxin and digitoxin from the foxglove plant *Digitalis*. Even though the positive effects of cardiac glycosides are well established, dose-dependent toxicity remains an issue (Ehle et al., 2011). Bufadienolides are present in very low amounts in plants and are prominent in animals such as the toad (*Bufo*), fireflies (*Photinus*), and snake (*Rhabdophis*) (Steyn and Van Heerder, 1998).

## Glycosylated Lipids

### Diacylglycerols

**FIGURE 2:**
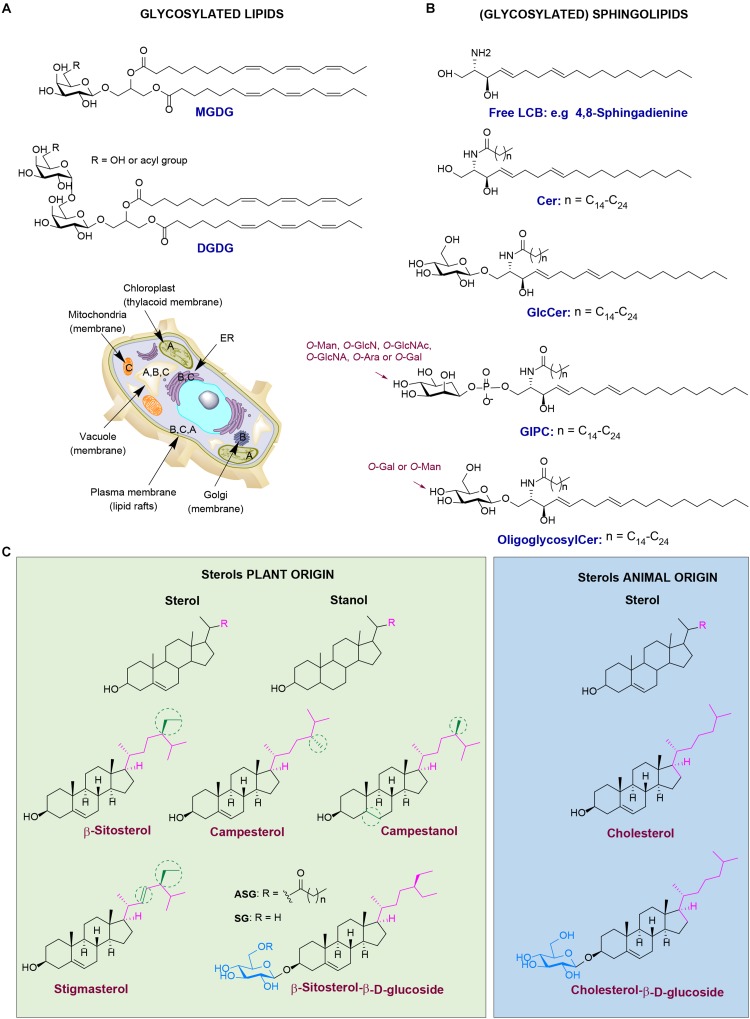
Different classes of plant lipids and their localization in the plant cell. **(A)** Chemical structures of plant glycosylated lipids: monogalactosyldiacylglycerol (MGDG) and digalactosyldiacylglycerol (DGDG). **(B)** Chemical structures of plant (glycosyl)sphingolipids: long-chain base (LCB) 4,8-sphingadienine, ceramide (Cer), glucosylceramide (GlcCer), glucosylinositol phosphoryl ceramides (GIPC) and oligoglycosylceramide. **(C)** Chemical structures of plant sterols (green) *versus* the animal counterparts (blue). Plant cell image illustrating the localization of lipid classes: **(A)** stands for MGDG and DGDG, **(B)** for plant (glycosyl)sphingolipids, and **(C)** for plant sterols.

Plants contain diverse glycosylated lipids. Galactosylated diacylglycerols are ubiquitous glycolipids in plants. They are predominantly found in photosynthetic tissues, such as the leaf. In particular, chloroplast thylakoid membranes contain high quantities of monogalactosyldiacylglycerol (MGDG) and digalactosyldiacylglycerol (DGDG) (see Figure 2A for the chemical structure and cellular localization) (Hölzl and Dörmann, 2019). For instance, MGDG and DGDG account to 36% and 20%, respectively, of spinach chloroplast glycerolipids. Less abundant are the acidic sulfoquinovosyldiacylglycerol (5%), and other glycerophospholipids (Wintermans, 1960). Of note, the existence of acylated MGDG (acylMGDG) has also been documented by Nilsson et al. (2015) and their concentration is increased during environmental stresses such as frost. Glucosylated diacylglycerols are far rarer than galactosylated counterparts in plants and animals. 1,2-Di-*O*-acyl-3-*O*-β-D-glucopyranosyl-*sn*-glycerol has however been found in rice bran (Holst, 2008).

### Sphingolipids

Glycosylated sphingolipids (glycosphingolipids) in which sugar(s) are attached to ceramide are very abundant in animal cells. Distinct sphingolipids exist in plants (see Figure 2B for the chemical structure and cellular localization). Plants produce, like animals, the simple glycosphingolipid glucosylceramide (GlcCer) where glucose is β-linked to a phytoceramide that differs from animal ceramide in the composition of the sphingosine moiety [also referred as long-chain base (LCB)] (Spassieva and Hille, 2003; Pata et al., 2010; Ali et al., 2018; Huby et al., 2019). Another glycosylated sphingolipid in plants is glucosylinositol phosphoryl ceramide (GIPC) (Ali et al., 2018). Whereas in animals the major sphingosine base is C18 LCB, in plants over nine different LCBs have been identified. In addition, the presence of dienes in the plant sphingosine bases is rather common (Pruett et al., 2008; Ali et al., 2018). Plant LCBs can occur in very low concentrations in cells, but they are mainly present as backbones of ceramides that further contain linked fatty acid chains (C16–C26). The ceramides are next glycosylated forming GlcCer and/or GIPCs (Ali et al., 2018). In plants, elongation of glucosylceramide to oligoglycosylceramides occurs with either mannosyl or galactosyl units (Lynch and Dunn, 2004). In the common mannosyl series in higher plants, up to four mannosyl units may be added via β1→4 linkages. Oligoglycosylceramides are present in the endoplasmic reticulum (ER), Golgi apparatus, vacuole membrane, and plasma membrane. Sphingolipids and their glycosylated forms together with plant sterols are important constituents of the lipid rafts of the plasma membrane (Msanne et al., 2015). The complex GCIPC are considered as the equivalents of complex glycosphingolipids like gangliosides in animal cells (Gronnier et al., 2016; Cacas et al., 2016). They play important roles in regulation of cellular processes, such as cell wall formation, programmed cell death, drought and salt tolerance (Ali et al., 2018).

#### Phytosterols (Plant Sterols and Stanols)

Phytosterols, also known as xenosterols, are essential components of plant cells that are predominantly found in cell membranes (Hartmann, 1998). They include plant sterols and stanols (saturated sterols without double bonds in the sterol ring) (see Figure 2C for chemical structure and cellular localization). Their chemical structure consists of a sterol body; a cyclopentano-perhydro-phenanthrene ring system (formed by four rigid rings) with a hydroxyl group at position C-3 and a side chain attached to the carbon C-17 (Figure 2C). Differences in the nature of the side chain gives a plethora of diverse sterols in plants, accounting to more than 260 different ones, as described over the last decades (Vanmierlo et al., 2015). The most abundant phytosterols in human diet are β-sitosterol, campesterol, campestanol, and stigmasterol. Their structure is similar to the structure of the mammalian sterol, cholesterol. Phytosterols structurally differ from cholesterol only at the length and saturation of their aliphatic side chain. For instance, campesterol has an additional methyl group at its side chain, at C-24 position (Mamode Cassim et al., 2019). It is important to mention that plants also contain small amounts of cholesterol (Hartmann, 1998). Phytosterols are mainly found in vegetable oils, seeds, and nuts and in less extent in fruits and vegetables (Amiot et al., 2011). Phytosterols play important roles in several biological processes. For instance, campesterol is found to act as a precursor at the biosynthesis of BRs, hormones that regulate plant growth, development and morphogenesis. In addition, β-sitosterol and stigmasterol are mainly involved in the maintenance of cell membranes, and together with sphingolipids, form the lipid rafts (Amiot et al., 2011; Ferrer et al., 2017). Phytosterols are also involved in responses to biotic and abiotic stresses (Ferrer et al., 2017). A characteristic example is the formation of stigmasterol in *Arabidopsis* leaves after inoculation with specific bacteria. In general, plant sterols play a key role in the innate immunity of plants against bacterial infections via regulating the nutrient efflux in the apoplast (Griebel and Zeier, 2010; Wang et al., 2012). In addition, tolerance to aluminum is shown to be influenced positively by the high sterol and low phospholipid contents in the root tip of plants. This results in a less negatively charged plasma membranes, tolerating better aluminum (Wagatsuma et al., 2014). At last, drought tolerance is also associated with sterol composition of the plants as studied via using the drought hypersensitive/squalene epoxidase 1–5 mutants in *Arabidopsis* (Posé et al., 2009).

### Glucosylated Phytosterols and Medicinal Properties

Conjugated forms of phytosterols occur. Examples are the steryl glycosides (SG) (sometimes referred to as sterolins) and their acylated forms; the acyl steryl glycosides (ASG). In plant SG, the sugar moiety, often a glucose, is attached at the C-3 hydroxyl group of the sterol. When the sugar moiety is further acylated with a fatty acid at the primary alcohol (C-6 carbohydrate numbering), ASG is formed (Grille et al., 2010; Nyström et al., 2012) (Figure 2C). The first glycosylated plant sterol to be purified was ipuranol from the olive tree in 1908. A few years later it was identified as β-sitosteryl-D-glycoside. ASGs were next discovered in lipid extracts of soybean seeds and potato tubers (Grille et al., 2010). Plant glycolipids occur in different amounts and in different composition among plant species even in different tissues from the same plant. High levels of SG and ASG occur in *Solanum* species, accounting to more than 50% of the total sterol levels (Nyström et al., 2012; Ferrer et al., 2017). SG and ASG levels are high in fruit, vegetable juices, beer, wine as well as in tomatoes and potatoes (Decloedt et al., 2017).

Steryl glycosides and ASGs play important roles in biological processes such as maintenance of the plasma membrane organization and they allow adaptive responses to environmental changes (Mamode Cassim et al., 2019). Several studies using forward and reverse genetic approaches have revealed the import role of SGs and ASGs in plants during different environmental stresses. An example is provided by transgenic *Arabidopsis* and *tobacco* plants, overexpressing a sterol GT from *W. somnifera*, showing increased tolerance toward salt, heat and cold. Furthermore, downregulation of the same gene product results in increased susceptibility to plant pathogens (reviewed in Ferrer et al., 2017). SGs and ASGs are also present in pollen and phloem sap of *Arabidopsis*. It has been hypothesized that SGs act as primers of cellulose synthesis (Ferrer et al., 2017). The attached sugar to the sterol increases drastically the hydrophilicity of phytosterols and might increase the ability to interact with proteins embedded in membranes as well as with other glycolipids in lipid rafts. The same has been proposed for the amphiphilic cardiac glycosides ouabain and digitalin (Tabata et al., 2008).

Phytosterols are nowadays widely used as food additives aiming to lower plasma LDL cholesterol and reduce cholesterol absorption in humans. Already in the early 1950s, intake of plant sterols was reported to reduce the total plasma cholesterol and LDL-cholesterol and cholesterol absorption efficiency (Peterson, 1951). After this, a vast number of studies and clinical trials demonstrated cholesterol-lowering effects of a phytosterol-rich diet. This led to the industrial production of phytosterol enriched food products such as margarines and yogurts (AbuMweis and Jones, 2008; Amiot et al., 2011; Trautwein et al., 2018; Nakano et al., 2019). It is generally accepted that 1–3 g of a daily dose of phytosterols leads to a 10–15% decrease of total cholesterol levels and also decreases plasma LDL-cholesterol (Patel et al., 2018; Nakano et al., 2019).

The metabolic response to phytosterols varies among individuals (Jones, 2015). This can be due to genetic differences, for example in genes encoding ApoE and CyP7A1. Of particular interest in this connection are also ATP-binding cassette (ABC) subfamily G member 5 (ABCG5) and member 8 (ABCG8), which are proteins involved in phytosterol transport. Mutations in ABCG5 or ABCG8 cause sitosterolemia, a devastating disease first described by Bhattacharyya and Connor (1974), Tada et al. (2018), Plat et al. (2019). The ABCG5 and ABCG8 proteins act as heterodimers, forming together with other proteins a functional sterol transport complex, and are expressed in hepatocytes, gallbladder epithelium, and enterocytes. ABCG5/ABCG8 excretes phytosterols and other xenosterols from cells, even better than cholesterol. Impaired ABCG5/ABCG8 leads to accumulation of phytosterols in the body causing macrothrombocytopenia, platelet dysfunction, liver disease, cholesterol accumulation with xanthoma formation and atherosclerosis (Silbernagel et al., 2013; Patel et al., 2018).

Little is still known on the impact of SGs and ASGs on the human body. The β-sitosterol-β-D-glycoside (BSSG) is relatively abundant in the human diet (Decloedt et al., 2017). Both β-sitosterol (BSS) and its glycosylated form are found in human plasma and tissues in very low levels, at 800–1,000 less compared to endogenous cholesterol (Pegel, 1997). It has been shown that upon chronic high intake plant sterols accumulate in the brain (Vanmierlo et al., 2012; Saeed et al., 2015). Even though the mammalian blood brain barrier (BBB) is not, or very poorly, permeable to cholesterol, studies have shown that phytosterols like sitosterol and campesterol are able to cross the BBB in mice.

β-Sitosterol and BSSG are proposed to exert beneficial anti-inflammatory actions and to reduce mild hypercholesterolemia (Bouic et al., 1999). Bouic et al. (1999) observed that administering a mixture of BSS/BSSGs to marathon runners had a positive effect on the immune system under physical stress conditions. On the other hand, BSSGs were also found to be toxic to motor neurons *in vitro* (Tabata et al., 2008). The exposure to high amounts of BSSGs amounts present in the seed of the cycad tree (*Cycas micronesica*) has been speculated to underly the historical high prevalence of the neurodegenerative disease amyotrophic lateral sclerosis–parkinsonism dementia complex (ALS–PDC) at the island of Guam (Tabata et al., 2008). Indeed, feeding of BSSGs to rats is found to cause several neurological signs and defects resembling those occurring in Parkinson disease patients, such as α-synuclein aggregates, motor abnormalities and striatal dopamine loss (Shen et al., 2010; Van Kampen et al., 2014; Kampen et al., 2015; Franco et al., 2018). Van Kampen et al. (2014) successfully induced Parkinsonism in Sprague Dawley rats by feeding them with BSSG for 4 months.

## Absorption and Metabolism of Plant Glycoconjugates

Knowledge on the absorption and metabolism of individual plant glycoconjugates is warranted to better understand their mechanism of action. It appears that upon ingestion the fate of individual glycoconjugates may fundamentally differ.

### Uptake of Glycosylated Flavonoids

For decades it was widely believed that prior to uptake in the body glycosylated flavonoids, such as quercitrin, rutin, and robinin, common components of human diets, were first deglycosylated by intestinal glycosidases and bacterial enzymes in the intestine (Griffiths and Barrow, 1972; Bokkenheuser et al., 1987). Only the produced aglycones would be partially absorbed in the large intestine (Walle, 2004). Following uptake, flavonoids are glucuronidated, *O*-methylated or sulfated in the liver. Part of them are subsequently excreted into the bile and undergo enterohepatic cycling to finally being eliminated by renal excretion (Murota and Terao, 2003). An example of such metabolism is that of the flavanone hesperidin, mainly found in citrus fruits such as oranges and lemons. Hesperidin (hesperetin with a linked rutinoside moiety) is absorbed as the aglycone hesperetin, after removal of the glycose moiety by intestinal bacteria. However, rapid absorption of α-glucosylated hesperidin (G-hesperidin) containing an additional linked α-glucosyl moiety has been observed, possibly due to its high water solubility (De Souza et al., 2016). It has become apparent that deglycosylation of some flavonoids may not depend on intestinal bacteria. Saliva has been also suggested to play a role in the hydrolysis of the dietary flavonoids. Browning et al. (2005) performed a study on different glycosylated flavonoids like quercitrin, rutin, isoquercitrin, spiraeoside, genistin, naringin, and phloridzin (Browning et al., 2005). Their findings suggest that saliva enzymes may hydrolyze the glycosylated flavonoids, in particular, glucosides. For instance, quercetin-4′-*O*-glucoside and genistein-7-*O*-glucoside, found in high amounts in dietary products, are rapidly hydrolyzed in the oral cavity. Saliva is also suggested to already hydrolyze quercetin glucosides (Hirota et al., 2001).

Dietary glycosylated anthocyanins, such as cyanidin-3-*O*-glucoside and cyanidin-3,5-*O*-diglucoside, are absorbed in intact form (Miyazawa et al., 1999). Quercetin glycosides are known to be taken up in the small intestine via the sodium dependent glucose transporter SGLT1 (Hollman et al., 1995; Walgren et al., 1998, 2000). Phloridzin, the glucoside of the flavonoid phloretin, was also found to be transported by SGLT1 (Walle and Walle, 2003). Takahashi et al. (2019) recently observed that intestinal absorption of galactosylated-cyanidin is inferior to that of the glucosylated one. Of note, absorbed glycosylated anthocyanins may pass the BBB and reach different brain regions like the cortex and hippocampus (Milbury et al., 2002; Milbury and Kalt, 2010; Zhang et al., 2019). Phloridzin and other flavonoid glycosides (quercetin and genistein) have also been identified as substrates for efflux by the multidrug resistance-associated protein transporters MRP1 and MRP2 (Walle and Walle, 2003). Thus, dietary glycosylated anthocyanins seem to manage to reach visceral tissues and the brain by hijacking glucose transporters and are actively removed by MRPs.

### Uptake of (Glycosylated) Phytosterols

In the lumen of the intestine the poorly water-soluble phytosterols are incorporated into micelles that allow close contact with the surface of enterocytes (Gylling et al., 2014). Next, phytosterols are thought to be internalized by the mucosal intestinal cells via the Niemann–Pick C1-Like1 (NPC1L1)-transporter. Subsequently, plant sterols are re-secreted into the lumen of the intestine via ABCG5/ABCG8 transporter complex (as discussed in section “Glycosylated Lipids”). In the liver, the ABCG5/ABCG8 complex mediates efflux of plant sterols into bile (Gylling et al., 2014). Plant sterols manage to pass the BBB and therefore potentially may influence brain function (Jansen et al., 2006; Vanmierlo et al., 2012). This notion raises considerations regarding excessive consumption of olive oil containing high amounts of plant sterols. The poor solubility of phytosterol in both water and oil limits absorption. Esterification of phytosterols increases their solubility in oil and margarine (Ostlund, 2004). Regarding glycosylated phytosterols it is clear that these reach tissues, including the brain (see section “Glycosylated Lipids”). Relatively little is, however, known with respect to transporter proteins involved in the uptake glycosylated sterols. They have been reported to be absorbed intact and exert as such their effects (Lin et al., 2009, 2011).

## Plant β-Glucosidases and Glucosyltransferases

### Classification of Glycosidases

All domains of living organisms contain multiple glycoside hydrolases (GHs, glycosidases). These enzymes play a variety of functions, including the lysosomal metabolism of glycolipids in animals, the catabolism of cell wall polysaccharides in plants and biomass conversion in microorganisms (Leah et al., 1995; Ketudat Cairns and Esen, 2010). More than 160 GH families have been listed in the Carbohydrate Active EnZymes (CAZy) database using a classification system based on amino acid sequence and secondary structure similarities (Henrissat, 1991; Ben Bdira et al., 2018). This classification system is regularly updated and new families are continuously discovered. Additionally, the enzymes are classified based on their reaction mechanism, according to the stereochemical outcome of the hydrolytic reaction, into inverting or retaining enzymes (Koshland, 1953; Sinnott, 1990). Moreover, glycosidases are also classified as exo- or endo-enzymes, depending on their ability to cleave at the end or in the middle of a carbohydrate chain.

Plants contain numerous CAZy-encoding genes, more than any other organism. For instance, *Arabidopsis* contains over 400 different genes encoding glycosidases (Husaini et al., 2018). This complexity stems from gene duplications and has likely been promoted by the increasingly complex plant cell wall structure, as described for *Arabidopsis* by Bowers et al. (2003). Some proteins, based on homology designated as glycosidases or glycosyltranferases might have further evolved to act on different types of substrates or to fulfill other non-enzymatic functions (Coutinho et al., 2003). An example is the soybean hydroxyisourate hydrolase. Even though the enzyme has a highly conserved retaining β-glucosidase active site, it catalyzes the hydrolysis of 5-hydroxyisourate (Raychaudhuri and Tipton, 2003). Therefore, caution when talking about plant glycosidases and GTs is necessary.

Particularly ubiquitous in plants are β-glucosidases. Most plant β-glucosidases (E.C.3.2.1.21) are mainly classified in the glycoside hydrolase family 1 (GH1) of the CAZy database. However, some plant β-glucosidases are grouped in GH families 5 and 30. They all fall in GH Clan A, and contain similar (β/α)_8_-barrel structures. They consistently share an active site with two catalytic residues (Morant et al., 2008; Ketudat Cairns and Esen, 2010). Their main activity, even though it is not restricted, accounts to the hydrolysis of the (β-glucosidic bond between carbohydrates or between a sugar and an aglycone moiety.

Plant β-glucosidases play a number of important biological roles. For instance, they are involved in cell wall degradation during endosperm germination (Leah et al., 1995). The enzymes together with other plant and microbial glycosidases and glycanases degrade the plant cell wall, leading to formation of intermediates for cell wall lignification (Dharmawardhana et al., 1995; Escamilla-Treviño et al., 2006). Over different enzymes have been reported taking a part in this process (Mohnen, 2008). Furthermore, β-glucosidases are involved in activation of phytohormones (Kristoffersen et al., 2000; Lee et al., 2006). They participate in plant defense mechanisms via activating several chemical defense compounds, like phytohormones, flavonoids and cyanogenic glucosides (Nisius, 1988; Poulton, 1990; Suzuki et al., 2006; Morant et al., 2008). For instance, a cyanogenic glucosidase (linamarase) from cassava and white clove is able to cleave glucosides from glucosylated cyanosides releasing toxic HCN as a defense mechanism (Oxtoby et al., 1991; Hughes et al., 1992). In addition, a β-glucosidase from maize was found to be active toward cytokinin-*O*-glucosides and kinetin-N3-glucoside, releasing active cytokinin (Brzobohaty et al., 1993). Furthermore, they are reported to release volatiles like flower scents from their glycoside storage forms (Sarry and Günata, 2004). Due to the high number of different plant glucosides, it is very likely that plant glucosidases play other roles that are yet to be discovered.

### Catalytic Mechanism of Glucosidases

Two carboxyl-exposing residues in the active site of both inverting and retaining β-glucosidases enzymes take part in the hydrolysis of the glycosidic bond (Koshland, 1953). In the case of inverting enzymes, these two groups are separated at a distance of 6–12 Å, whereas in retaining enzymes, this is ∼5 Å. The inverting reaction is a single step reaction; a direct displacement of the aglycone, where one carboxylic group is acting as the base and it activates a water molecule that hydrolyzes the glycosidic bond through a nucleophilic attack at the anomeric center (Guce et al., 2010) and at the same time, the second carboxylic acid facilitates the departure of the leaving group via acid catalysis. On the contrary, retaining glycosidases employ a double displacement mechanism (Koshland, 1953). The reaction initiates with the nucleophilic attack to the anomeric center, resulting in a glycosyl-enzyme covalent intermediate. Then, the deprotonated carboxylate acts as a base and deprotonates a water molecule, that now plays the role of a nucleophile, to hydrolyze the covalent intermediate giving the reaction product. The transfer of a released sugar from a substrate to an acceptor other than a water molecule is called transglycosylation, and has been observed for several retaining glycosidases (Sinnott, 1990; Hehre, 2001). The acceptor molecules can be sugars, as in the case of chitotriosidase (Aguilera et al., 2003), but also retinol or sterol in the case of glucocerebrosidase, the human β-glucosidase (Vanderjagt et al., 1994). Akiyama et al. (2013) and Marques et al. (2016a) reported the use of glucosylceramide as sugar donor in the formation of cholesterol glucoside via β-glucosidase mediated transglucosylation. Several examples of transglycosylation activity of plant and bacterial glycosidases have also been reported (Crout and Vic, 1998; Morant et al., 2008).

### Glycosyltransferases

The CAZy database currently contains 110 GTs. In plants, GTs have many functions, for example in the biosynthesis of glycosylated metabolites, oligosaccharides, polysaccharides (cellulose, hemicelluloses, and pectins among others), and glycoproteins in the plant cell membrane (Hansen et al., 2012). The polysaccharides and other glycans are mainly synthesized by GTs (EC 2.4.x.y). Most GTs (Leloir GTS) transfer a sugar residue from an activated nucleotide sugar to a specific acceptor molecule, with high specificity for the sugar donor and the acceptor substrates (Breton et al., 2005). GTs are classified as retaining or inverting depending on whether glycosylation results in net retention or inversion of stereochemistry at the anomeric carbon of the donor substrate. GTs are classified in the CAZy database into families on the basis of amino acid sequence similarities (Cantarel et al., 2009). Two major folds of structures of nucleotide–sugar-dependent GTs solved to date are observed, termed GT-A and GT-B (Hansen et al., 2010). Many GT-Bs are independent of a metal ion for catalysis, whereas most GT-A enzymes contains a conserved DxD motif that coordinates the phosphate atoms of the nucleotide donors via coordination of a divalent cation, usually Mn^2+^ or Mg^2+^ (Breton et al., 2005). Besides GTs using sugar mono- or diphosphonucleotide donors, known as Leloir type GTs, two additional group of glucosyltransferases occur: non-Leloir-type GTs which employ sugar lipid phosphates, pyrophosphates or polyprenol phosphates as donors, and non-activated acyl-glucose dependent glucosyltransferases. This last group of enzymes are transglucosidases related to GH1 family hydrolases. One example of this is the rice β-glucosidase Os9BGlu31 that uses glucopyranosides as well 1-*O*-acyl glucose esters as sugar donors in synthetic reactions (Luang et al., 2013; Komvongsa et al., 2015).

## New Tools to Explore “Plant” Glycosidases; Activity Based Probes (Abps) for Retaining Glycosidases

Detailed knowledge on the reaction mechanism of retaining glycosidases has allowed the generation of ABPs, a new class of versatile research tools (for a recent review see Wu et al., 2019).

### ABPs, Principles and Applications Through Time

The idea to exploit covalent inhibitors of active enzymes as ABPs was firstly put forward for esterases by Ostrowski and Barnard (1961). The concept was further pioneered by Cravatt et al. (2008) for several enzyme classes. Now, ABPs have been designed for kinases, proteases, serine hydrolases, lipases and glycosidases (Cravatt et al., 2008; Witte et al., 2010, 2011; Serim et al., 2012; Baggelaar et al., 2013; Kallemeijn et al., 2014; Willems et al., 2014b; Kuo et al., 2018).

In the case of retaining glycosidases, the use of irreversible inhibitors optimally mimicking the oxocarbenium ion-like transition state of the target enzyme has promoted the design of diverse ABPs. Wicki et al. (2002) and Goddard-Borger et al. (2010) first proposed the use of fluorinated inhibitors for targeting human β-glucosidase. Chauvigné-Hines et al. (2012) generated difluoromethylphenyl aglycone, *N*-halogenated glycosylamine, and 2-deoxy-2-fluoroglucoside ABPs to identify reactive glycosidases in the cellulosomal secretome of *Clostridium thermocellum.* Their study identified a wide number of cellulases, xylanases, hemicellulases, and CAZys. More specific, they were able to identify both inverting and retaining enzymes that were listed in different GH families. Some of them play an important role in the degradation of cellulose: GH9 (inverting) cellulase enzymes, a number of GH5 (retaining) endoglucanases, and GH10 and GH11 (retaining) xylanases (Chauvigné-Hines et al., 2012).

**FIGURE 3:**
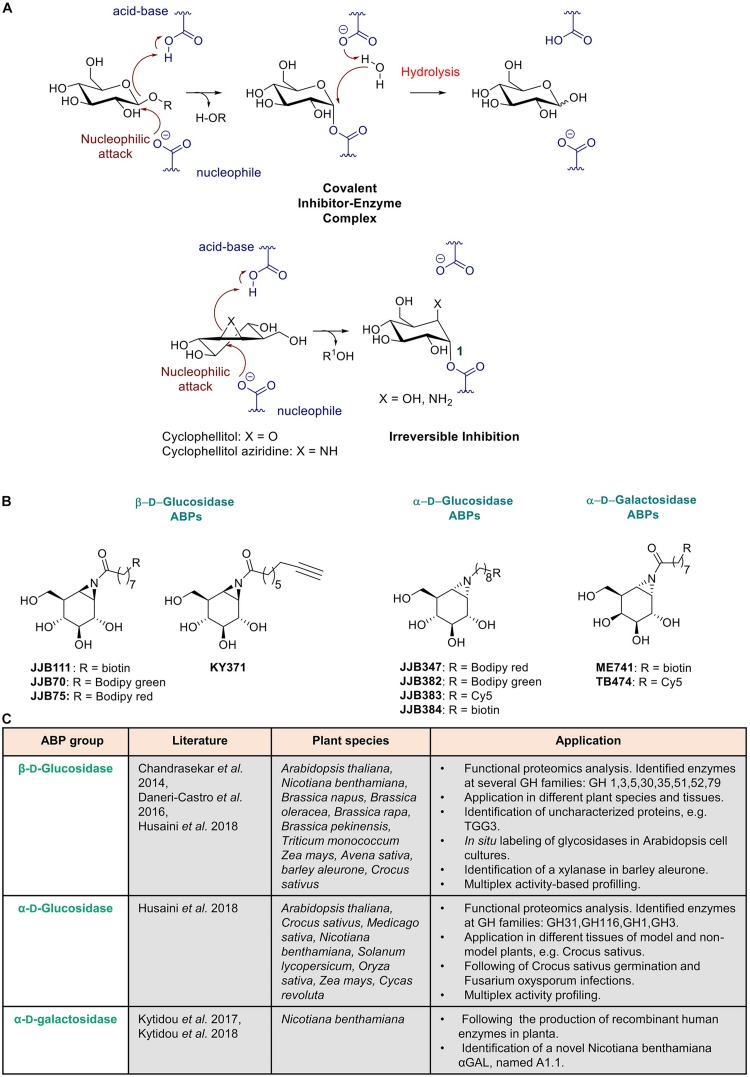
Activity based probes mechanism and use in plant science. **(A)** Mechanism of aziridine and epoxide activity-based probes. **(B)** Structures of ABPs used in plant science. **(C)** Summary of published ABPs used in plant research. Presented are key publications describing specific applications in plant species.

The first retaining (β-glucosidase for which a high affinity and highly selective ABP was designed has been human glucocerebrosidase. A decade ago, Witte et al. (2010) demonstrated that cyclophellitol based ABPs irreversibly bind and enable identification of GBA (human lysosomal β-glucosidase; glucocerebrosidase) in complex biological samples, such as cell lysates, cell cultures, and laboratory animals. This ABP has meanwhile found important applications in studies on Gaucher disease, an inherited lysosomal storage disorder caused by GBA deficiency. Its use for diagnostic purposes holds great promise (Aerts et al., 2011). The glucosyl-configured cyclophellitol ABP binds covalently to the catalytic nucleophile, Glu-340, of the human GBA enzyme (Figure 3A). In addition to the cyclophellitol sugar, the probes contain a reporter group, that can be a fluorophore and/or biotin, attached via a linker to the C-6 position of the functionalized cyclophellitol, enabling the visualization and/or identification of active enzyme molecules (Witte et al., 2010).

Next, a different class of ABPs was designed by Overkleeft for retaining β-glucosidases based on the use of cyclophellitol-aziridine scaffolds (Kallemeijn et al., 2012) (Figures 3A,B). Subsequently, by variation of the configuration of cyclophellitol-aziridine a series of new probes were developed for other retaining glycosidases. Detection of active α-D-galactosidase (Willems et al., 2014a), β-D-galactosidase (Marques et al., 2016b), α-L-fucosidase (Jiang et al., 2015), α-D-glucosidases (Jiang et al., 2016), β-D-glucuronidase (Wu et al., 2017), α-L-iduronidase (Artola et al., 2018) and α-L-arabinofuranosidases (McGregor et al., 2020) was achieved with these types of ABPs. Through changing the reporter group into a biotin, the probes can be used for streptavidin-mediated enrichment, followed by proteomics identification of labeled proteins using LC-MS/MS (Jiang et al., 2016). Meanwhile, many research applications for glycosidase ABPs have been found. The amphiphilic nature of glycosidase ABPs enables them to penetrate cells and organisms, allowing *in situ* labeling of active enzyme molecules in lysosomes. Intravenous infusion of mice with ABPs resulted in specific labeling of GBA in various tissues (Kallemeijn et al., 2012). More recently, the same ABPs were employed to very sensitively visualize active GBA molecules in lysosomes of cells by correlative light electron microscopy (CLEM) (Van Meel et al., 2019). Visualization of GBA in the brain of mice was also achieved after intracerebroventricular administration of the ABP (Herrera Moro Chao et al., 2015). Application of ABPs in studies with zebrafish models of GBA enzyme deficiency have been recently described (Artola et al., 2019; Lelieveld et al., 2019). In addition, β-D-glucuronidase ABPs have recently been employed in fecal samples to study the anticancer Irinotecan drug toxicity driven by gut microbial enzymes (Jariwala et al., 2020).

### ABPs in Plant Science

Plant scientists interested in hydrolases have to deal with a huge number of genes encoding potential glycosidases. Moreover, transcript expression levels do not always correlate with actual active enzyme levels. Therefore, methods for conveniently monitoring distinct active glycosidases are in great demand to establish the functional proteome of different plant species. ABPs provide a novel toolbox for this purpose which has been successfully applied in plant science during the last decade (van der Hoorn and Morimoto, 2016). Enzymes of several families have been identified and functionally characterized using ABPs, such as serine hydrolases (Kaschani et al., 2009), papain-like cysteine proteases (Richau et al., 2012) and cysteine proteases (Lu et al., 2015). In addition, retaining glycosidases of different plant species have been characterized and identified (Chandrasekar et al., 2014; Husaini et al., 2018; Kytidou et al., 2018). The use of ABPs as functional tool for studying retaining glycosidases in plants was first reported by the group of Chandrasekar et al. (2014). The chemical structures of ABPs used in this research are depicted in Figure 3B. An overview is provided in Figure 3C of key publications describing specific applications of ABPs in research using several plant species.

The (β-D-glucose configured cyclophellitol-aziridines **JJB70** conjugated with a BODIPY green fluorophore and **JJB111** conjugated with a biotin was combined with proteomic analysis of targeted protein to study glycosidases in *Arabidopsis thaliana* total leaf samples and in *N. benthamiana* apoplast samples (Chandrasekar et al., 2014). **KY371** probe, which does not contain any reporter group, was applied as competitor to ensure that protein labeling was specific. The investigation revealed that the aziridine type β-glucosidase ABPs present a broad activity, enabling identification of not only β-glucosidases but also myrosinases, xylosidases, and galactosidases. Importantly, very high (micromolar) concentrations of ABP were used in the study which favors detection of different classes of glycosidases. The identified proteins are members of seven different retaining glycosidase families (Chandrasekar et al., 2014). Of note, no cellulases were identified. The main identified proteins were the β-thioglucoside glucohydrolase TGG2 (68-kDa signal) and TGG1 (75-kDa signal). Both enzymes are myrosinases and catalyze the conversion of glucosinolates during attack of invaders. Interestingly, yet uncharacterized glycosidases, one of which was previously classified as pseudogene (TGG3), were identified in the study. An investigation with the ABP of secreted proteins by *N. benthamiana* cells led to the identification a wide range of putative xylosidases, galactosidases, glucanases, and heparanase. In addition, *in situ* labeling of active glycosidases present in *Arabidopsis* cell cultures revealed that ABPs (**KY371 and JJB70**) can penetrate living plant cells and therefore can be fortuitously also used to generate knock out models. Furthermore, van der Hoorn and Morimoto (2016) explored the presence of glycosidases in different (tissues of) plant species like *Brassica napus, Brassica oleracea, Brassica rapa, Brassica pekinensis, Triticum monococcum, Zea mays, Avena sativa*, and *Nicotiana benthamiana* via in-gel imaging (Chandrasekar et al., 2014) (see Figure 3C).

Next, Daneri-Castro et al. (2016) used ABPs to identify and characterize enzymes that are secreted by the aleurone layer during barley germination and are induced or not by gibberellic acid (GA). They employed different ABP classes and thus were able to identify putative aleurains, cathepsin-B-like proteases and serine hydrolases. **JJB70** ABP, targeting active retaining glycosidases, was used to demonstrate the presence of a putative xylanase in barley aleurone by competing the labeling with xylose (Daneri-Castro et al., 2016). More recent, ABPs targeting (α-glucosidases have been used in investigations on Arabidopsis and saffron crocus (Crocus sativus) (Husaini et al., 2018). Interestingly, using the α-glucosidase ABP (**JJB383**) evidence was obtained that during stigma development in saffron glycosidases are involved in the conversion of picrocrocin into safranal. Furthermore, during Fox infection the enzyme AGLU1 was detected to be present in the apoplast. In the same study, parallel analysis of both α- and β-glucosidases was performed, enabling simultaneous identification of different enzyme classes (Husaini et al., 2018). In conclusion, ABPs find very broad applications in investigations on plant metabolism and physiology.

Activity-based probes have also already been successfully used to monitor recombinant active enzyme during transient expressions in *N. benthamiana* plants. Human α-galactosidases have been produced in *N. benthamiana* leaves and in HEK293 cell cultures (Kytidou et al., 2017). Recombinant protein was detected and quantified using the α-galactosyl configured cyclophellitol-aziridine **TB474** containing a Cy5 fluorophore (Kytidou et al., 2017). This detection method is superior to western-blot since it allows selective detection of active enzyme molecules. Based on previous investigations, it might be concluded that ABPs can be applied as an easy quantitative method to follow the production of biopharmaceuticals in recombinant systems. In addition, the use of the biotinylated ABP, **ME741**, enabled the identification of a novel *N. benthamiana* galactosidase, named A1.1. The enzyme was then overexpressed in *N. benthamiana* leaves, purified and further biochemically characterized. One of the most important findings was that A1.1 proves to be able to hydrolyze human glycosphingolipids *in vitro* and *in situ* and might find applications in the treatment of Fabry disease, caused by deficiency of the human α-galactosidase (Kytidou et al., 2018). Therapeutic application of a plant enzyme, discovered with an ABP, for treatment of a human metabolic disease can be thus be envisioned (Figures 3B,C).

The specificity of ABP labeling of glycosidases can be fine-tuned by the assay conditions (e.g., variation of pH), concentration of probe and use of competitive inhibitors. The ABPs can in principle be used to visualize glycosidases during physiological processes of interest such as plant development, seed germination, cell wall formation and different responses to biotic and abiotic stresses.

## Production of Therapeutic (Human) Glycosidases in Plants

### History

Production of biopharmaceutical proteins in plants is undertaken since the early 2000s. It gained great attention due to the advantages in economy, safety and scalability with several examples of *in planta* recombinant protein productions over the last decades (Westerhof et al., 2014; Wilbers et al., 2017). The first plant-produced protein was the human growth hormone, in 1986, in *tobacco* cell cultures (Fischer et al., 2004). After that, a vast number of different proteins have been produced *in planta*, demonstrating the viability of such methods for industrial and pharmaceutical uses (Hidalgo et al., 2018; Kopertekh and Schiemann, 2019). A recent example is the large scale production of the drug ZMapp in 2014 in *tobacco* leaves for use against Ebola virus (Yao et al., 2015). This review focuses on the production of therapeutic human glycosidases in plants for the treatment of lysosomal diseases.

### Lysosomal Enzymes and Their Production in Plants

**FIGURE 4:**
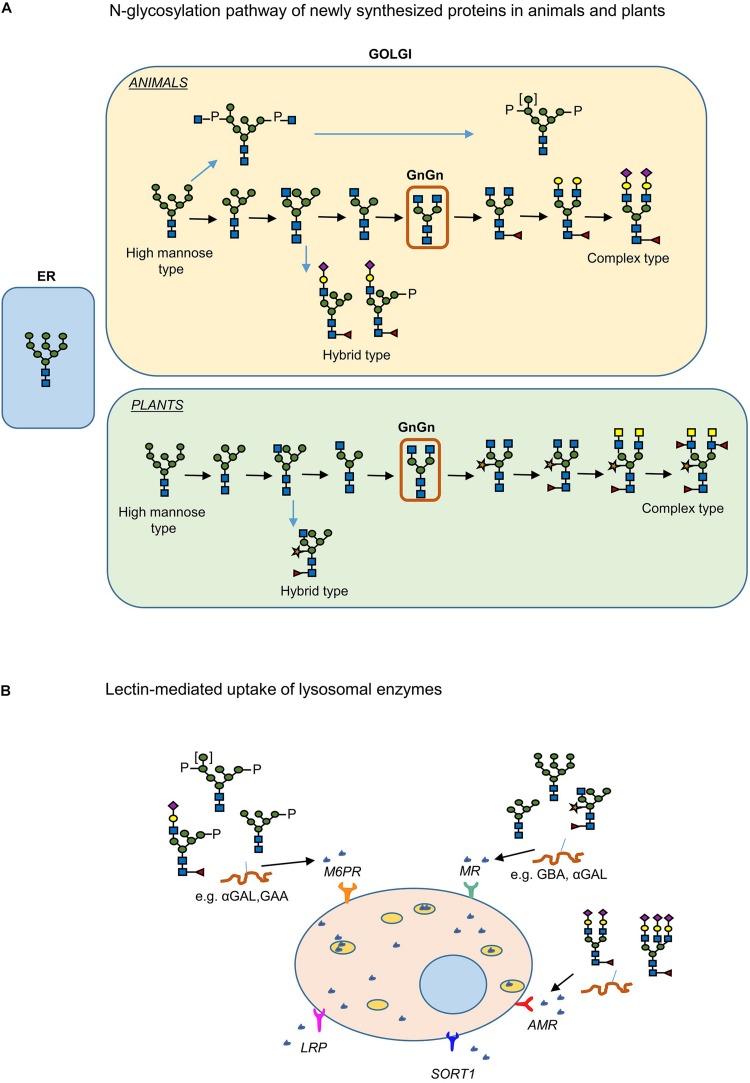
Plant and human *N*-glycosylation pathways: importance in production of pharmaceuticals. **(A)** *N*-linked glycosylation pathway of proteins produced in plants and humans. **(B)** The lectin mediated uptake of recombinant enzymes from cells for the treatment of LSDs.

Lysosomal diseases are inherited metabolic diseases caused by dysfunction of lysosomal hydrolases. A possible therapy for these diseases involves replacement of deficient enzymes by their normal equivalents. Supplementation of patient cells is envisioned following intravenous administration (infusion) of recombinant enzyme. This concept was developed and pioneered by Brady and is known as enzyme replacement therapy (ERT) (Brady, 2003; Desnick and Schuchman, 2012; Aerts and Cox, 2018). The uptake of the recombinant enzyme preparations is usually mediated by mannose receptors (MR) which are present in the surface of the targeted cells and also via mannose-6-phosphate receptors (M6PR) and the asialoglycoprotein receptor (Ashwell-Morell receptor; AMR) (Figure 4) (Coutinho et al., 2012; Shen et al., 2016; Tian et al., 2019). Examples of such disorders and their corresponding impaired glycosidase are Gaucher disease and β-glucocerebrosidase (GBA), Fabry disease and α-galactosidase (GLA), Pompe disease and α-glucosidase (GAA) and Krabbe disease and β-galactocerebrosidase (GALC).

The production of the lysosomal human GBA enzyme in carrot cells by Shaaltiel et al. (2007) can be considered as a hallmark in the production of pharmaceutical glycosidases in plants for the treatment of lysosomal diseases. The plant produced enzyme, ELELYSO (taliglucerase alfa) (Protalix BioTherapeutics), was approved by the FDA in May 2012. The enzyme is targeted to the vacuoles of carrot cells via the insertion of a chitinase (“GLLVDTM”) vacuole signal peptide. The recombinant enzyme has high mannose terminal *N*-glycans that mediate uptake by macrophages (Shaaltiel et al., 2007). It was firstly demonstrated that the plant specific *N*-glycans on the plant produced recombinant GBA are not immunogenic, a crucial finding. Production of human GBA enzyme in transgenic, complex-glycan-deficient, *Arabidopsis* seeds, has also been achieved (He et al., 2011). The produced enzyme in the latter case does not contain any targeting signal to the vacuole. Active GBA has recently also been produced in root cultures of *Nicotiana tobacco* (Naphatsamon et al., 2018). Finally, GBA enzyme with high mannose *N*-glycans (gnt1-GBA) was produced in mutant rice (Jung et al., 2019).

Another human lysosomal hydrolase produced in plants is α-galactosidase A. The production of this enzyme for therapy of Fabry disease was firstly accomplished in tobacco cell cultures in 2015 (Kizhner et al., 2015; Ruderfer et al., 2018; Schiffmann et al., 2019). The protein, pegunigalsidase alfa, PRX-102 (Protalix BioTherapeutics), is currently in clinical trials and it is produced in large scale at ProCellEx plant cell-based protein expression platform (Ruderfer et al., 2018). Interestingly, the recombinant protein is chemically modified with a bifunctional PEG polymer to form stable dimers. Such modification improves the enzyme’s stability in plasma and its pharmacokinetic properties (Ruderfer et al., 2018). PRX-102 is found superior in stability than the current available recombinant enzymes for treatment of Fabry disease (agalsidase beta, Fabrazyme and agalsidase alpha, Replagal) which are both produced in mammalian cell culture systems. Of note, no M6P residues are present in the N-linked glycans of pegunigalsidase alfa. A mannose enriched human α-galactosidase A was produced in 2016 in moss cell cultures (Shen et al., 2016). The enzyme was found to be effectively targeted through the MR pathway to defective organs such as heart and kidney. α-Galactosidase was also produced transiently in *N. benthamiana* leaves for research purposes (Kytidou et al., 2017). In parallel, in the same platform human α-*N*-acetylgalactosaminidase and a mutated form with increase α-galactosidase activity was generated (Tajima et al., 2009; Kytidou et al., 2017). Finally, a modified fragment of human α-glucosidase (GAA) has been produced in plant chloroplasts to induce tolerance against human GAA in Pompe disease patients receiving ERT (Su et al., 2015). Future investigations will demonstrate clinical benefit of this approach.

### *N*-Glycoengineering

The feasibility to manipulate and humanize the *N*-glycosylation pathway of plants offers a great advantage in the production of biopharmaceuticals (Castilho and Steinkellner, 2012; Bosch et al., 2013). *N*-Glycosylation of glycoproteins in plants and mammals is identical up to the formation of the vital intermediate “GlcNAc_2_Man_3_GlcNAc_2_” *N*-glycan (GnGn structure) in medial-Golgi apparatus (Figure 4A) (Gomord et al., 2010). Further modification of the GnGn structure takes place in mammals in *trans*-Golgi apparatus, resulting in complex and highly heterogenic *N*-glycan structures whereas in plants further modifications are not as frequent and mainly include the addition of β(1,2)-xylose and α(1,3)-fucose residues at core GnGn structure (Gomord and Faye, 2004). Additionally, high mannose, paucimannosidic structures and also Lewis-^*X*^- epitopes are frequently observed in *N*-glycans of plant glycoproteins. Even though core fucosylation may occur in mammals this involves addition of α(1,6)-linked fucose residues (Bosch et al., 2013). Several examples of *N*-glycoengineering plants to reach a human like *N-*glycan profile have been reported and reviewed (Castilho and Steinkellner, 2012; Bosch et al., 2013). Using reverse genetics (CRISPR/cas9 knock out, RNAi methods), plants were generated lacking endogenous activities such as the ones of β (1,2)-xylosyltransferase and core α (1,3)-fucosyltransferase responsible for attaching plant-specific residues to core glycan structures (Jansing et al., 2019). This was first accomplished in *Arabidopsis thaliana* plants followed by *Nicotiana benthamiana*, *Lemna minor*, and the moss *Physcomitrella patens* and rice cells (Strasser et al., 2004; Schähs et al., 2007; Loos et al., 2011; Castilho and Steinkellner, 2012; Bosch et al., 2013).

During ERT of patients suffering from glycosidase deficiencies, intravenously infused recombinant enzymes are endocytosed via lectin-mediated pathways. In the case of Gaucher disease, ERT is highly successful since the recombinant enzyme is targeted efficiently to macrophages (the primary storage cells) via MR-mediated uptake (see above and Figure 4B). In other LSDs, however, several cell types are affected and need to be supplemented with therapeutic enzyme. For this reason, use of the ubiquitous M6PR uptake is envisioned and recombinant enzyme with a high M6P content in their *N*-glycans are produced (Schiffmann et al., 2001; Eng et al., 2001; Kroos et al., 2012; Ferraz et al., 2014). Thus, the *N*-glycan profile of the therapeutic enzyme plays a key role in its availability, targeting and bioactivity. The α-galactosidase produced in moss by Shen et al. (2016) is claimed to be endocytosed by many cell types via mannose-lectin mediated uptake. The enzyme has high mannose *N*-glycans that lack the plant specific α (1,3)-fucose and β (1,2)-xylose residues (Shen et al., 2016).

## Abps: Linking Retaining Glycosidases With Small Compound Interactors of Their Catalytic Pockets

The previous sections of this review largely focused on glycosylated metabolites on the one hand and retaining glycosidases on the other hand. In addition, the design and application of ABP reacting in a mechanism-based manner with the catalytic nucleophile of specific glycosidases was introduced. In this section, the use of ABPs to ‘bridge’ glycosidases with interacting small compounds is discussed.

**FIGURE 5:**
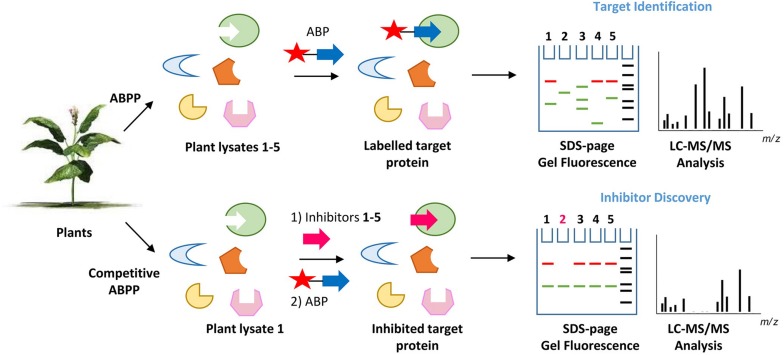
Principle of activity-based protein profiling for target identification and competitive activity-based protein profiling for the screening of glycosidase inhibitors. The competition of ABP labeling of a glycosidase by agents interacting with its pocket (inhibitors, substrates) can be conveniently and sensitively assessed. there is no need for a pure enzyme, when visualizing labeled glycosidase using SDS-PAGE and fluorescence scanning.

The great value of ABPs to identify, purify and characterize glycosidases from various plants has been addressed in the previous sections. Some of these enzymes might find future applications as drugs or in industrial processes. Another application for ABPs warrants discussion. By virtue, ABPs can be also used to identify small compounds that interact with the catalytic pocket of the reactive glycosidase. Such interactors, (substrates, inhibitors), will compete with the ABP for occupancy of the pocket and the subsequent labeling of the enzyme (Figure 5). This concept can for example be exploited to screen different plant extracts or selected compounds (glycosylated plant sterols and flavonoids) for interaction with (plant or human) glycosidases as revealed by the competition of labeling of the glycosidase with the corresponding ABP. Proof of principle for such screens has already been obtained. In this manner, inhibitors have been identified for the human non-lysosomal glucosylceramidase GBA2, an enzyme that is difficult to purify to homogeneity in active form. Lahav et al. (2017) used a fluorescent polarization activity-based protein profiling (ABPP) assay where they successfully screened a library of 350+ iminosugars for potential GBA2 inhibitors using a lysate of cells over-expressing GBA2 and the appropriate glucosidase ABP (Lahav et al., 2017). In the same manner, complex biological samples can be screened on the presence of potential substrates for a glycosidase competing with ABP labeling. One further application along the same line is the use of cell-permeable ABP labeling of a glycosidase to identify the *in situ* inhibition of a target glycosidase by an administered inhibitor. An example of such application is provided by a recent study identifying in intact cells and zebrafish the β-glucosidase target engagement of conduritol B-epoxide and cyclophellitol analogs (Kuo et al., 2019).

In conclusion, ABPs will not only be of value to study their target glycosidases but also interactors of their catalytic pockets.

## Summary and Perspectives

Natural plant-derived glycosides are used for various therapeutic purposes. Increased knowledge of beneficial/toxic effects is warranted. This is particularly relevant for plant sterols for which beneficial and potentially harmful effects have been reported. Better insight is needed regarding the biological effects, bioavailability and metabolism of glycosylated sterols prior to any clinical use in prevention/treatment of diseases. This may also hold for other plant metabolites. The therapeutic value of infusion of glycosidases in treatment of inherited deficiencies in man has been demonstrated for a number of diseases. In recent years, production of such glycosidase increasingly occurs in plant platforms that offer several advantages. Importantly, the *N*-glycan composition of plant-produced recombinant enzymes can be very well controlled using genetically modified plants. The ubiquitous plant glycosidases themselves might conceivably find therapeutic applications in humans and might have potential to treat inherited glycosidase deficiencies in man (Kytidou et al., 2018). Overall, ABPs may help to identify plant glycosidases of interest.

## Author Contributions

KK and JA wrote the manuscript with input from MA and HO. KK together with MA conceived and designed the figures with input from JA and HO.

## Conflict of Interest

The authors declare that the research was conducted in the absence of any commercial or financial relationships that could be construed as a potential conflict of interest.
